# Supplementary material for: A Healthy Diet Is Not More Expensive than Less Healthy Options: Cost-Analysis of Different Dietary Patterns in Mexican Children and Adolescents
Source: Nutrients. 2021 Oct 29;13(11):3871. doi: 10.3390/nu13113871 (PMC8624608; doi:10.3390/nu13113871)
Supplement: Supplementary file 1 [file nutrients-13-03871-s001.zip › nutrients-1387328-supplementary.pdf]

Supplementary Table S1. Food grouping used in the dietary pattern analysis

Main group	Basis for placing a food item	Food groups	Food items
Grains	Culinary use	1. Corn tortilla	Corn tortilla.
		2. Mexican food	Pozole, memela, quesadilla, sope, taco.
	Proportion of fiber	3. Whole grains	Whole bread, oatmeal, linseed, all Bran, multi bran, multigrain.
		4. Refined grains	White bread, wheat tortilla, rice, corn flakes, honey crunch, other cereals.
	Specific nutrient profile	5. Pastries	Pastries.
		6. Desserts	Cookies, cakes.
		7. Snacks	Potato chips, crackers.
Vegetables	Proportion of fiber	8. Fresh vegetables	Cauliflower, spinach, lettuce, carrots, tomato, nopal, onion, corn, cabbage, pea, green bean, chili, hot pepper, beet, mixed vegetables.
	Culinary use	9. Tomato juice	Tomato juice.

	Proportion of starch	10. Potatoes	Potatoes.
Fruits	Proportion of fiber	11. Fresh Fruits	Banana, prune, peach, apple, orange, avocado, grapes, strawberry, melon, watermelon, mango, tangerine, pear, mamey, zapote, papaya, pineapple, guava, prickly pear.
	Frequency of consumption	12. Orange juice	Orange juice.
Meats	Frequency of consumption	13. Eggs	Eggs.
		14. Poultry	Chicken with or without skin.
	Specific nutrient profile	15. Red meat	Pork, beef or lamb.
		16. Processed meat	Sausage, bacon, ham.
		17. Fish and other sea food	Canned tuna fish, sardine, fresh fish, octopus, and squid.
Dairy	Specific nutrient profile "proportion of fat"	18. Low-fat dairy products	Skim or low-fat milk, low-fat yogurt.
		19. High fat dairy products	Whole milk, chocolate milk, cream, high fat yogurt, cream

			cheese, other cheese, ice cream.
Legumes	Frequency of consumption	20. Legumes	Lentils, dry beans.
Fat	Specific nutrient profile “proportion of fat and type of fat”	21. Oils and nuts	Peanut, walnut, almond, pistachios, vegetable oils.
		22. Butter	Margarine, butter, mayonnaise, animal fats.
Sugar	Frequency of consumption “proportion of sugar”	23. Sweet food and sugars	Sugar, chocolate, candies, jam, honey, “ate”, jelly.
		24. Soft drinks	Soft drinks.
		25. Other sweetened	Sweetened beverages.
		26. Low-energy drink	Low-energy carbonated beverages.
Alcohol	Relative frequency of consumption	27. Alcohol	Coolers, spirit, wine, beer, brandy, whisky, tequila, rum, hard liquor, pulque.
Other beverages	Relative frequency of consumption	28. Natural water<br>29. Energetic drinks	Water.<br>Gatorade and other energetic drinks.

Supplementary Table S2. Unit cost of the 133 foods in the food frequency questionnaire.

Product	Purchase unit	Unit cost of product		Conversion of purchase unit to ration	Portion 4th edition SMAE	SMAE unit portion
Whole milk	1L	MX$	20.05	0.240	240	ml
Skim milk	1L	MX$	20.10	0.240	240	ml
Semi-skimmed milk	1L	MX$	20.50	0.240	240	ml
Milk with sugar	1L	MX$	8.00	0.240	240	ml
Cream cheese	1Kg	MX$	39.70	0.045	45	gr
Oaxaca cheese	1Kg	MX$	173.80	0.030	30	gr
Manchego cheese	1Kg	MX$	223.75	0.025	25	gr
Sour cream	1Kg	MX$	13.55	0.015	15	ml
Ice cream (with milk)	1L	MX$	30.00	0.099	99	gr
Sorbet (without milk)	1L	MX$	18.00	0.040	40	gr
Yoghurt	1L	MX$	25.63	0.227	227	gr
Danone/danonino	1L	MX$	35.50	0.250	250	gr
Fermented products (Yakult, etc.)	1L	MX$	70.00	0.240	240	ml
Margarine	1L	MX$	57.00	0.004	4	gr
Butter	1L	MX$	115.66	0.006	6	gr
Egg	1Kg	MX$	36.70	0.050	50	gr

Chicken	1Kg	MX$	39.90	0.040	40	gr
Bacon	1Kg	MX$	223.50	0.008	8	gr
Sausage	1Kg	MX$	90.00	0.061	61	gr
Ham	1L	MX$	123.00	0.042	42	gr
Liver	1L	MX$	46.90	0.030	30	gr
Chorizo (pork sausage)	1L	MX$	64.00	0.015	15	gr
Beef	1L	MX$	139.00	0.030	30	gr
Pork meat	1L	MX$	80.00	0.040	40	gr
Tuna	1L	MX$	154.00	0.033	33	gr
Sardine	1L	MX$	137.00	0.036	36	gr
Fish	1L	MX$	644.00	0.040	40	gr
Octopus	1L	MX$	124.63	0.025	25	gr
Pork rind	1L	MX$	154.50	0.012	12	gr
Barbacoa (barbecued lamb)	1L	MX$	450.00	0.050	50	gr
Turkey breast	1Kg	MX$	196.75	0.032	32	gr
Pork ham	1Kg	MX$	187.00	0.042	42	gr
Mayonnaise	1Kg	MX$	64.10	0.005	5	gr
Oil	1L	MX$	36.00	0.005	5	ml
Lard	1kg	MX$	35.50	0.004	4	gr

Cauliflower	1kg	MX$	33.50	0.200	200	gr
Corn	1kg	MX$	38.50	0.083	83	gr
Potato	1kg	MX$	24.00	0.095	95	gr
Spinach	1kg	MX$	120.00	0.120	120	gr
Calabazas	1kg	MX$	20.70	0.110	110	gr
Lettuce	1kg	MX$	42.50	0.141	141	gr
Tomato	1kg	MX$	23.30	0.120	120	gr
Tomato puree	1kg	MX$	20.00	0.122	122	gr
Cactus	1kg	MX$	20.20	0.140	140	gr
Avocado	1kg	MX$	27.90	0.058	58	gr
Pumpkin flower	1kg	MX$	40.60	0.132	132	gr
Beet	1kg	MX$	17.50	0.043	43	gr
Onion	1kg	MX$	38.10	0.058	58	gr
Green bean	1kg	MX$	36.60	0.063	63	gr
Pea	1kg	MX$	71.60	0.053	53	gr
Broad bean	1kg	MX$	25.00	0.032	32	gr
Lentil	1kg	MX$	20.00	0.035	35	gr
Bean	1Kg	MX$	34.00	0.035	35	gr
Vegetable soup	1L	MX$	15.00	0.240	240	ml

Salsa	1L	MX$	33.00	0.115	115	gr
Pepper	1Kg	MX$	25.00	0.040	40	gr
Chickpea	1Kg	MX$	27.00	0.035	35	gr
White bean	1Kg	MX$	42.00	0.035	35	gr
Banana	1Kg	MX$	20.00	0.080	80	gr
Plum	1Kg	MX$	55.70	0.198	198	gr
Peach	1Kg	MX$	65.50	0.174	174	gr
Apple	1Kg	MX$	49.00	0.138	138	gr
Orange	1Kg	MX$	14.00	0.242	242	gr
Orange juice	1L	MX$	21.00	0.120	120	ml
Grape	1Kg	MX$	57.50	0.126	126	gr
Strawberry	1Kg	MX$	65.30	0.204	204	gr
Melon	1Kg	MX$	16.95	0.271	271	gr
Watermelon	1Kg	MX$	8.00	0.160	160	gr
Mango	1kg	MX$	53.50	0.207	207	gr
Tangerine	1kg	MX$	16.50	0.180	180	gr
Pear	1kg	MX$	30.50	0.095	95	gr
Mamey	1kg	MX$	37.00	0.137	137	gr
Tuna	1kg	MX$	13.50	0.250	250	gr

Zapote	1kg	MX$	38.15	0.056	56	gr
Papaya	1kg	MX$	23.90	0.140	140	gr
Pineapple	1kg	MX$	14.85	0.124	124	gr
Guava	1kg	MX$	25.50	0.135	135	gr
Raisin	1Kg	MX$	102.00	0.020	20	gr
Peanut	1kg	MX$	54.50	0.012	12	gr
Nut	1kg	MX$	155.00	0.009	9	gr
Almond	1kg	MX$	130.00	0.012	12	gr
Cake	1kg	MX$	38.00	0.028	28	gr
Pastries	1kg	MX$	55.00	0.017	17	gr
Cookie	1kg	MX$	112.00	0.021	21	gr
Chocolate	1kg	MX$	56.00	0.015	15	gr
Jam	1kg	MX$	76.50	0.017	17	gr
Corn tortilla	1kg	MX$	15.00	0.030	30	gr
Flour tortilla	1Kg	MX$	39.25	0.028	28	gr
Bread roll	1kg	MX$	55.00	0.020	20	gr
Sandwich bread	1Kg	MX$	34.75	0.027	27	gr
Whole-grain sandwich bread	1kg	MX$	35.25	0.025	25	gr
Cracker	1Kg	MX$	38.00	0.016	16	gr
Rice	1kg	MX$	38.00	0.020	20	gr

Oatmeal	1Kg	MX$	21.00	0.020	20	gr
Pasta soup	1kg	MX$	46.00	0.050	50	gr
Fried foods	1Kg	MX$	115.00	0.019	19	gr
Al pastor tacos	1Kg	MX$	190.00	0.150	150	gr
Memela, quesadilla, etc.	1Kg	MX$	35.00	0.150	150	gr
Pozole	1L	MX$	55.00	0.310	31	gr
Bran	1Kg	MX$	35.00	0.054	54	gr
Boxed cereal	1Kg	MX$	55.60	0.015	15	gr
Cola drink	1L	MX$	13.00	0.092	92	ml
Flavored soda	1L	MX$	13.00	0.089	89	ml
Diet soda	1L	MX$	13.00	0.355	355	ml
Fruit “agua fresca” with sugar	1L	MX$	15.00	0.500	500	ml
Sugarless cup of tea	1L	MX$	10.00	0.240	240	ml
Sugarless cup of coffee	1L	MX$	24.50	0.240	240	ml
Atole cup with milk	1L	MX$	22.00	0.240	240	ml
Atole cup without milk	1L	MX$	22.00	0.240	240	ml
Cup of chocolate with milk	1L	MX$	22.00	0.240	240	ml
Cup of chocolate without milk	1L	MX$	22.00	0.240	240	ml

Glass of wine	1L	MX$ 175.00	0.200	200	ml
Cup of beer	1L	MX$ 24.00	0.356	356	ml
Alcohol	1L				
Alcoholic drink (liquor)	1L	MX$ 60.00		55	ml
Natural fruit juice	1L	MX$ 19.50	0.080	80	ml
Industrialized fruit juice	1L	MX$ 18.35	0.125	125	ml
Vegetable juice	1L	MX$ 39.95	0.121	121	ml
Industrialized vegetable juice	1L	MX$ 20.00	0.125	125	ml
Natural water	1L	MX$ 12.00	0.240	240	ml
Mineral water	1L	MX$ 13.50	0.240	240	ml
Tea with sugar (industrialized)	1L	MX$ 25.00	0.240	240	ml
Tea with non-caloric sweetener (industrialized)	1L	MX$ 27.50	0.240	240	ml
Coffee with sugar	1L	MX$ 24.50	0.240	240	ml
Coffee with non-caloric sweetener	1L	MX$ 24.50	0.240	240	ml
Agua fresca with sugar	1L	MX$ 25.00	0.240	240	ml
Agua fresca with non-caloric sweetener	1L	MX$ 18.35	0.240	240	ml
Industrialized water with sugar	1L	MX$ 18.35	0.240	240	ml

Non-caloric industrialized water	1L	MX$	18.00	0.240	240	ml
Sports drink	1L	MX$	18.35	0.152	240	ml
Energetic drink	1L	MX$	18.35	0.078	78	ml
Sugarless frappé	1L	MX$	45.00	0.240	240	ml
Sugar frappé	1L	MX$	45.00	0.240	240	ml

MX= Mexican peso.
